# Evaluation of yield, yield components and some quality traits of tuber of potato (*Solanum tuberosum* L.) under different weed and nutritional management practices

**DOI:** 10.3389/fpls.2024.1495541

**Published:** 2025-01-06

**Authors:** Hooshmand Amjadi, Gholamreza Heidari, Sirwan Babaei, Zahed Sharifi

**Affiliations:** ^1^ Department of Plant Production and Genetics, Faculty of Agriculture, University of Kurdistan, Sanandaj, Iran; ^2^ Department of Soil Science, Faculty of Agriculture, University of Kurdistan, Sanandaj, Iran

**Keywords:** biological herbicide, foliar spraying, harvest index, seaweed extract, tuber-reducing sugars

## Abstract

Potato (*Solanum tuberosum L.*) production requires effective nutrient and weed management strategies to enhance tuber yield and quality while minimizing the environmental impact of chemical inputs. This study investigated the effects of various weed and nutrient management practices on potato tuber yield, yield components, and quality traits. The experiments were conducted over two years (2019–2020) at the University of Kurdistan’s research farm in the Dehgolan Plain, using a split-plot based on randomized complete block design with four replicates. Main plot treatments included a control (no fertilizer), complete chemical fertilizer (based on soil testing), foliar application of edible mushroom powder, and seaweed extract. Subplot treatments focused on weed control methods, consisting of a control (no weed control), chemical herbicides (metribuzin + paraquat), a biological herbicide, and manual weeding (weed-free). The highest tuber yield was achieved with the combination of metribuzin and paraquat herbicides alongside manual weeding in the complete fertilizer regimen, yielding 25 and 23.2 t ha⁻¹, respectively. Treatments with edible mushroom powder resulted in a 32% increase in tuber counts but a 21% decrease in individual tuber weights compared to the control. Tuber quality was significantly affected, with phosphorus concentration peaking at 0.26% under the complete fertilizer regimen, whether paired with manual weeding or the biological herbicide. Additionally, reducing sugars were highest in the complete fertilizer and chemical weed management treatments, indicating potential implications for tuber processing quality. These findings suggest that an integrated approach combining complete chemical fertilizer application with effective weed control maximizes both total yield and nutrient concentrations in potatoes. The results provide valuable insights for developing sustainable potato production practices that balance yield optimization with environmental stewardship.

## Introduction

Crops face various environmental stresses during growth, impacting their health and productivity. These stresses are generally categorized as biotic, such as pests, diseases, and weeds, or abiotic, including factors like drought, heat, and frost (Amjadi et al., 2019). Among biotic stresses, weeds are estimated to contribute to a 9% reduction in global crop yields (Neve et al., 2018). Due to their aggressive growth and ability to rapidly exploit resources, weeds outcompete crops for essential nutrients, water, and light. This competition results in decreased yields, increased production costs, and reduced farmer incomes (Silva et al., 2018). As global food demand continues to rise—projected to increase by 50% over the next century—addressing weed management effectively becomes a critical priority for sustainable agriculture (Juraimi et al., 2013). Controlling weeds is essential for maintaining optimal crop productivity and ensuring food security (Neve et al., 2018).

Potato (*Solanum tuberosum* L.), is among the world’s most important food crops, cultivated on over 17 million hectares globally, with an annual production exceeding 370 million tons (Gustavsen, 2021). As a major staple crop with industrial and nutritional significance, potatoes play a vital role in supporting food security due to their high yield potential and rich nutrient content (Singh et al., 2020). However, potato cultivation faces challenges from weed infestations that significantly affect yield and tuber quality. Weeds compete with potato plants early in the season, and without effective management, this can lead to substantial reductions in yield and nutritional quality of the tubers (Mondani et al., 2011; Yadav et al., 2015). Chemical herbicides are commonly used to combat weed pressure in potato fields, and they have shown high efficacy in weed suppression, often achieving over 90% reduction in weed density (Abdallah et al., 2021). Conventional control methods, such as the use of metribuzin and paraquat, are widely recognized for their efficacy in managing a broad spectrum of weed species in potato cultivation (Andidi, 2023). This combination has become a standard approach due to its reliable weed suppression across various cropping systems. Nonetheless, reliance on chemical herbicides has raised environmental concerns due to their persistence and toxicity, as well as health risks for those involved in their application (Elena, 2012; Mesi et al., 2012; Cherry et al., 2018). Additional research investigating the environmental pollution potential of specific herbicides in onion fields underscored the necessity for caution in herbicide application to mitigate negative environmental impacts (Rosculete et al., 2019; Adeli et al., 2023). Increasing herbicide resistance further underscores the need for alternative, sustainable weed management approaches (Moradi et al., 2023; 2024; Naghib Alsadati et al., 2020; Sharifi Kaliani, et al., 2024).

One promising solution lies in the integration of biological herbicides, which include aqueous extract of weeds like *Sonchus oleraceus* L. (sow thistle) (Singh et al., 2003), plant pathogenic microorganisms and microbial phytotoxins, as a means of suppressing weeds with minimal environmental impact (Pacanoski, 2015). To be effectively integrated into weed management, biological herbicides must be economically feasible, capable of causing high mortality in target weeds, and safe for both the environment and human health (Kremer, 2019; Hershenhorn et al., 2016). Their formulation often involves plant pathogens that have been selectively modified to increase efficacy (Hershenhorn et al., 2016). Additionally, bio-based products, such as foliar extracts from edible mushrooms and seaweed, are increasingly valued for their roles in enhancing crop tolerance to stress, which can improve both crop health and productivity (Rai et al., 2021; Kumar et al., 2021; Radi and Banaei-Moghaddam, 2020).

The foliar application of edible mushroom powder, rich in essential nutrients and bioactive compounds, can enhance plant growth by supplying amino acids, vitamins, and polysaccharides, which support plant health and resilience (Fedeli et al., 2024). These bioactive compounds promote enzymatic activity and improve plant metabolism, potentially increasing crop tolerance to environmental stresses and enhancing nutrient uptake (Godoy et al., 2021). Similarly, seaweed extracts contain plant growth hormones, such as cytokinins, auxins, and gibberellins, which can stimulate root growth, improve chlorophyll production, and increase plant resilience to stress factors (Mughunth et al., 2024; Deolu-Ajayi et al., 2022). Seaweed extracts also offer a source of essential minerals and antioxidants that can improve tuber quality and yield, while reducing the need for chemical inputs (Ali et al., 2021a).

Given the adverse effects of weed competition on potato yield and quality, along with the environmental implications of chemical herbicide use, this study seeks to evaluate the impact of integrating weed management strategies with various nutrient treatments, including foliar applications of edible mushroom powder and seaweed extracts, on potato production. Specifically, we aim to investigate how different combinations of weed and nutrient management affect the yield, yield components, and quality characteristics of potato tubers. By exploring the combined effects of these bio-based nutrient treatments with weed management, this research contributes to identifying sustainable agricultural practices that minimize environmental impact while supporting crop productivity.

## Materials and methods

### Study site and soil characteristics

**Figure 1:**
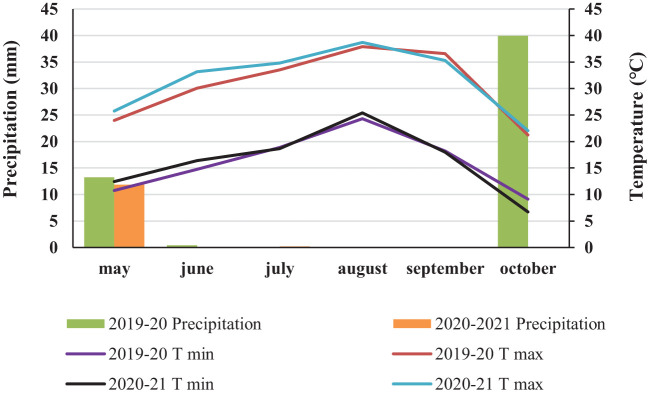
Average meteorological characteristics of the experimental area during crop seasons (2019-2020 and 2020-2021).

To investigate the changes in yield and yield components of potato tubers under different weed and fertilization management methods, a two-year experiment was conducted in 2019 and 2020 at the University of Kurdistan research farm, located in the Dehgolan Plain (35°18’ N, 47°18’ E) with an elevation of 1866 meters above sea level. The information related to the meteorological statistics of the region during two crop years is presented in **Figure 1**. **Table 1** presents some of the physical and chemical properties of the soil where the experiment took place. The soil temperature and moisture regimes of the soil where the experiment took place, are Mesic and Xeric, respectively. According to the World Reference Base (WRB, 2022), soil taxonomy system (USDA, 2003), the soil reference groups in the research area were Cambisols and Cacisols. To obtain the characteristics of the soil, 3 independent composite samples (each consist of 3 subsamples), were taken from the topsoil (0 – 25 cm depth). The soil samples were immediately transported in clean plastic bags to the laboratory. In the laboratory, the soil samples were left to air-dry and then passed through a 2.0 mm sieve to remove additional coarse materials. Then main physical and chemical properties of the soil samples were determined by standard methods as follows. Particle-size distribution was determined using the hydrometer method as reported by Gee and Bauder (1986). Soil pH and electrical conductivity (EC) were measured on a 1:2 soil/water (w/v) mix by a pH-meter and a conductometer (both by Metrohm Pty Ltd., Herisau, Switzerland), respectively. Total organic carbon (TOC) was measured by Walkley and Black (1934) chromic acid wet oxidation method, while the total organic matter (TOM) was calculated as: TOM% = TOC% × 1.724 (Nelson and Sommers, 1996). Total organic nitrogen (TON) was determined by the Kjeldahl method (Bremner and Mulvaney, 1982). Available potassium was extracted by 1M NH_4_COOH buffered at pH 7.0, then determined by the flame photometer (Model BWB-1, Technology, UK Ltd.). Aavailable phosphorus was extracted by 0.5 M NaHCO_3_ buffered at pH 8.5, and quantified by a spectrophotometer (Cary 50, Varian Australia Pty Ltd. Mulgrave, Victoria), according to Murphy and Riley (1962). The available boron was extracted with hot water (Berger and Truog, 1944), and quantified by a spectrophotometer. Available concentrations of Fe and Zn were quantified by 1.0 M NH_4_NO_3_ extraction using a 10:25 soil:solution (W/V) after 2 h shaking (Ravikovitch et al., 1986), and quantified by atomic absorption spectrophotometry (Varian SpectrAA 220, Varian Australia Pty Ltd. Mulgrave, Victoria).

**Table 1:** Some physical and chemical characteristics of the soil in which the experiment was conducted.

Properties	Value
Sand (%)	16.1
Silt (%)	37.9
Clay (%)	46.6
Texture	Clay
pH	7.49
Electrical conductivity (dSm^-1^)	0.50
Organic carbon (%)	0.52
Organic matter (%)	0.90
Total nitrogen (%)	0.075
Available P (mg kg^-1^)	12.54
Available K (mg kg^-1^)	335
Available Fe (mg kg^-1^)	1.95
Available Zn (mg kg^-1^)	0.08
Available B (mg kg^-1^)	0.68

As shown in **Table 1**, the soils of the research area are alkaline in reaction (pH = 7.49), non-saline in salinity (EC = 0.50 dSm^-1^), and heavy in texture (clay = 46.6%). According to the threshold limit of 3.4% of organic matter (OM) for normal soils (Golchin, 1994), the soil samples were found to be deficient in OM content (OM = 0.90%). The amount of available phosphorus and potassium of this soil was 12.54 and 335.0 mgkg^-1^, respectively. Based on Soil and Water Research Institute of Iran (SWRI) guideline, a plant available soil P concentration below 12 mg kg^−1^ and a K concentration below 350 mg kg^−1^ were not adequate for satisfactory tuber production, thus, the soil samples were found to be deficient in the K content (Malakouti and Ghaibi, 2000). The amount of available iron, zinc, and born was 1.95, 0.08, 1.31 and 0.68 mgkg^-1^, respectively (**Table 1**), which based on SWRI’s guideline, the soil samples were found to be deficient in the microelements content for satisfactory tuber production (Khavazi et al., 2014).

### Experimental design

**Figure 2:**
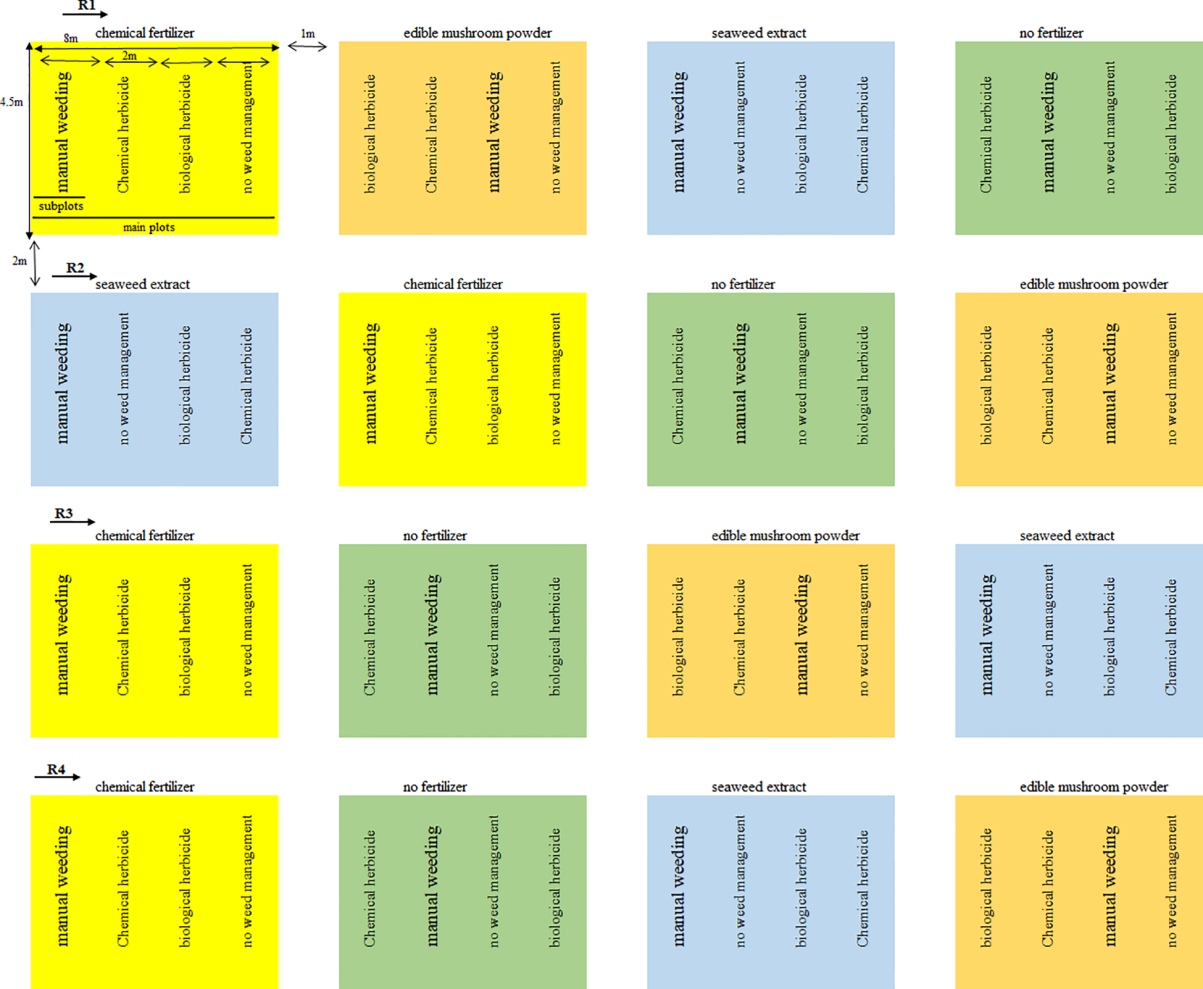
The experimental setup under the different treatment conditions.

The experiments were designed as a split plot in a randomized complete block design, with four replications. The experimental treatments included four fertilization methods serving as the main plots: chemical fertilizer based on soil test recommendations (F_1_), foliar application of edible mushroom powder (F_2_), foliar application of seaweed extract (F_3_), and a control with no fertilizer (F_4_). The subplots consisted of four weed control methods: manual weeding (H_1_), a combination of metribuzin and paraquat herbicide (H_2_), a biological herbicide (H_3_), and a control with no weed management (H_4_). **Figure 2** illustrates the experimental setup under the different treatment conditions.

Each main plot measured 5 meters in width and 8 meters in length, containing six planting rows. The rows were spaced 75 cm apart, with 22 cm between plants within each row. Additionally, there was a 1-meter spacing between main plots and a 2-meter distance between replications.

In order to apply fertilization treatments based on the results of soil tests before sowing the crop, urea (46% nitrogen) produced by domestic petrochemical companies, potassium sulfate (containing 20% potassium and 25% sulfur), and triple superphosphate (46% phosphorus) were used.

For the foliar spraying treatments, a seaweed extract (including 8% nitrogen, 12% alginic acid, 25% seaweed extract, 10% potassium, 0.5% micronutrients, and 40% organic matter) and edible mushroom powder were used. The seaweed extract and edible mushroom powder were first mixed with distilled water and then applied as foliar sprays in two stages (early vegetative growth and just before entering the flowering stage) in the afternoon (to reduce evaporation and increase absorption efficiency). The dosage for the seaweed extract was applied based on the usage instructions at a ratio of 3 per thousand. Similarly, for the edible mushroom powder, a ratio of 3 per thousand was used in the two stages of early vegetative growth and just before entering the reproductive stage.

It is worth mentioning that all treatments were carried out with distilled water to reduce experimental error and provide uniform conditions for all treatments. The control treatment did not use any fertilizers, either in foliar application or in soil application.

Chemical herbicide treatment involved applying a combination of metribuzin (700 grams per hectare) from Mashkafam Fars Company and paraquat (3 liters per hectare) from the National Agricultural Chemicals Company. This treatment was administered to the designated subplots three to four days prior to potato emergence, during the early morning hours. For weeding, manual removal of all weeds in the relevant plots was conducted once a week. In the case of biological herbicide application, a 4% concentration of the prepared extract was sprayed during the cooler hours of the day (at 7:30 AM), three days before the potatoes emerged. Additionally, a control treatment was included, which involved plots that received no herbicide treatment whatsoever.

Land preparation and planting took place in June, which is the typical planting period for potatoes in the region. The potato variety ‘Sprite’ was treated with Mancozeb fungicide to protect against fungal diseases. It was planted on June 3 in the 2019 and on June 17 in the 2020. using a semi-automatic tuber drill at a density of 60,606 plants per hectare. Other agricultural practices, including irrigation and pest and disease management, were implemented in a manner that did not negatively impact yield. To manage fungal diseases, iprodione+carbendazim was applied at a rate of 1/1000 prior to the flowering stage.

### Fertilization and weed control treatments

The application of seaweed extract and edible mushroom powder, diluted in distilled water, was conducted through foliar spraying at two key stages: early vegetative growth and just before the flowering phase. This spraying was performed in the afternoon to minimize evaporation and enhance absorption efficiency. The dosage for the seaweed extract followed the manufacturer’s usage instructions, while a concentration of 3 grams per liter was used for the edible mushroom powder. To ensure consistency across treatments and minimize experimental error, all treatments were also foliar-sprayed with distilled water.

Additionally, the collection and preparation of plant materials for the production of biological herbicides took place in April and May of 2018 and 2019. Fresh foliage of the sow thistle (*Sonchus oleraceus* L.) was harvested, air-dried in the shade, ground into a fine powder, and stored in plastic bags in a refrigerator at 2 degrees Celsius until needed (Gomaa et al., 2014). To prepare the extract, 40 grams of the ground leaves of the sow thistle were soaked in 1 liter of distilled water at a temperature of 20 degrees Celsius for 24 hours. During this period, the mixture was shaken every few hours to ensure optimal extraction of the herbal components. After 24 hours, the plant extract was filtered through two layers of filter paper to eliminate plant residues and then centrifuged at 15,000 rpm for 15 minutes. The resulting supernatant was further filtered using Whatman filter paper (Gomaa et al., 2014).

### Harvesting and laboratory analysis

Harvesting was performed manually on October 20 from a 2 m² plot. The tubers from each treatment were collected in separate bags and weighed after being transported to the laboratory. The number of stems per plant was recorded by counting all visible stems at the time of harvest. Tuber density was measured by counting the total number of tubers harvested from a square meter. The average tuber density for each treatment was calculated, and statistical comparisons were made among the treatments to identify any significant differences. To determine the average number of tubers per plant, all tubers associated with each plant were counted at harvest. The average weight of the tubers was measured using a digital scale. Tubers from each treatment group were weighed, and the average weight per treatment was calculated. Potato yield was quantified by weighing the total tubers harvested from each plot and converting this to a standardized yield per hectare (t.ha^-1^). The harvest index was calculated as the ratio of the weight of the harvested tubers to the total plant biomass at the time of harvest.

Given that this timing coincides with the peak accumulation of tuber dry matter, samples of the potato tubers were taken. The samples were dried in an oven at 85 degrees Celsius for 48 hours before being weighed. Subsequently, the dried samples were ground into a powder for laboratory analysis. The starch content of all treatments was measured using the anthrone-sulfuric acid method with a spectrophotometer (double beam spectrophotometer model 6850 in front of England) set to a wavelength of 630 nm, following the protocol by (Mc-Cready et al., 1956). Tuber reducing sugars concentration was determined using the phenol-sulfuric acid method as outlined by (Dubois et al., 1956). A film photometer (device XP model of the British BMW company) was utilized to assess potassium levels, with data recorded in parts per million (ppm) and converted to percentage according to the standard curve established by (William, 2000). For phosphorus concentration, measurements were again taken in ppm at a wavelength of 550 nm and converted to percentage following Jones (2001). Additionally, nitrate concentration in the tubers was analyzed using the method described by Jones (2001).

### Statistical analysis

The data were subjected to the combined analysis of variance over years and the chi-square test was used to verify homogeneity of variance before combining data using SAS software version 9.4. Mean comparisons performed using the Least Significant Difference (LSD) test. Graphs were drawn by EXCEL software.

## Results

The results indicated that nutritional treatments and their interaction with weed control treatments significantly influenced all measured traits (p < 0.01) (**Table 2**). Specifically, the weed control treatments alone had a significant effect on the number of tubers per plant, as well as on tuber phosphorus and potassium concentrations, sugar and nitrate levels, and overall tuber yield. However, these treatments did not significantly affect other traits. Furthermore, interactions between the year and nutritional treatments, year and weed control treatments, and year with both nutritional and weed control treatments were not significant for any traits (**Table 2**). There was little difference in rainfall and temperature between the two years of the experiment. Both years exhibited similar climatic conditions (**Figure 1**).

**Table 2:** Combined variance analysis of yield and quality of potato tubers affected by different weed and nutritional management practices in two years.

S.O.V	DF	Number of stems per plant	The number of tubers on the harvest surface	Average number of tubers per plant	Average weight of tubers	Harvest index (%)	Tuber phosphorus concentration	Potassium concentration of the tuber	Tuber reducing sugars	The percentage of tuber starch	Tuber nitrate concentration	Yield (wet weight of tubers)
Year (Y)	1	2.8^*^	128^ns^	6.48ns	578^ns^	18.9^ns^	0.027^**^	4.68^**^	1.64^**^	204.9^**^	307.5^**^	48672^ns^
R/Year	6	0.21	214.5	3.05	186.2	9.04	0.001	2.87	0.9	9.3	95.3	2497024.1
Nutritional management (N)	3	2.5^**^	1279.28^**^	21.2^**^	5162^**^	55.74^**^	0.009^**^	0.27^**^	5.55^**^	671.9^**^	2776.7^**^	11394145.70^**^
Y × N	3	0.00178^ns^	0.0018^ns^	0.0012^ns^	0.00019^ns^	0.00014^ns^	0.000006^ns^	0.01^ns^	0.003^ns^	1.7^ns^	0.00001^ns^	0.0003^ns^
Error 1	18	0.63	38.8	2.02	182.8	8.53	0.002	0.02	0.01	3.02	0.1	1090468.3
Weed management (W)	3	1.05^ns^	896.4^ns^	24.1^**^	264.7^ns^	3.81^ns^	0.003^**^	0.08^*^	0.06^**^	2.54^ns^	148.4^**^	6375919.99^**^
N × W	9	2.26^**^	420.4^**^	13.3^**^	1036.1^**^	18.52^ns^	0.0006^**^	0.46^**^	0.19^**^	22.2^**^	44.00^**^	3400810.37^**^
W × Y	3	0.00301^ns^	0.0021^ns^	0.0021^ns^	0.00412^ns^	0.0018^ns^	0.000012^ns^	0.004^ns^	0.01^ns^	1.76^ns^	0.00012^ns^	0.0025^ns^
N × W × Y	9	0.00278^ns^	0.0023^ns^	0.00001^ns^	0.00381^ns^	0.0011^ns^	0.000006^ns^	0.006^ns^	0.018^ns^	1.82^ns^	0.000011^ns^	0.0032^ns^
Error 2	72	0.56	100.3	2.28	209.6	11.53	0.000068	0.02	0.012	2.06	0.3	647071
Coefficient of variation		21	18.5	19	21	4.27	3.5	4.59	4.9	3.34	0.3	22

ns, * and ** according to non-significance, presence of significant difference at 5 and 1 percent probability level.

### Yield components, yield and harvest index

The results showed that the highest number of stems per plant was achieved with the foliar application of edible mushroom (**Table 3**). Although there was no significant difference between this and the complete fertilizer treatment (based on soil tests) or foliar seaweed extract, all these treatments had significantly more stems than the control. The biological herbicide treatment also produced the highest stem count, significantly differing from the no-weed-management control (p < 0.05) (**Table 3**).

**Table 3:** Mean comparison for interaction of fertilizer and weed management treatments on some potato morphological traits and harvest index.

Traits					
Factors	Number of stems per plant	The number of tubers per unit area	Average number of tubers per plant	Average weight of tubers	Harvest index (%)
Fertilizer treatments					
F1	3.7 a	55.3 a	8.1 a	82.7 a	80.96 a
F2	3.76 a	59.18 a	7.9 a	59.63 b	80.08 a
F3	3.4 ab	56.6 a	8.31 a	65.5 b	77.80 b
F4	3.14 b	44.8 b	6.5 b	76.28 a	79.14 ab
LSD 5%	0.45	6.31	0.73	7.83	1.82
Weed management					
H1	3.43 ab	59.2 a	8.11 a	72 a	79.49 a
H2	3.59 ab	56.6 ab	8.55 a	68.7 a	79.43 a
H3	3.71 a	52.9 b	7.8 a	65.08 a	80.01 a
H4	3.29 b	47 c	6.53 b	69.3 a	79.14 a
LSD 5%	0.35	4.63	0.92	8.93	1.65

F1 complete chemical fertilizer (based on soil test results), F2 edible mushroom powder, F3 seaweed extract, F4 without fertilizer, H1 manual weeding, H2 metribuzin + paraquat herbicide, H3 biological herbicide, H4 without herbicide (in each column, common letters in nutrition treatments and weed management means no significant difference based on LSD test).

The foliar edible mushroom treatment yielded the highest tuber density, with an average of 59.18 tubers per square meter, though this was not significantly different from the other three fertilizer treatments. For tubers per plant, the seaweed extract foliar treatment led with an average of 8.31 tubers. However, this result was not significantly distinct from the complete fertilizer, foliar edible mushroom, or seaweed extract treatments, all of which performed similarly well. By contrast, the control without fertilizer had the lowest average, with only 5.6 tubers per plant, indicating that optimal nutrient supply supports favorable plant spread and development.

Weed management influenced tuber density, with manual weeding yielding the highest average at 59.2 tubers per m^2^, and the no-herbicide control treatment producing the lowest, at 47 tubers. All weed management treatments, apart from the herbicide-free control, were statistically similar. Fertilizer treatments also impacted average tuber weight: the complete fertilizer treatment, based on soil analysis, produced the highest average tuber weight of 82.7 g, while the foliar edible mushroom treatment resulted in the lowest average weight at 59.63 g.

**Figure 3:**
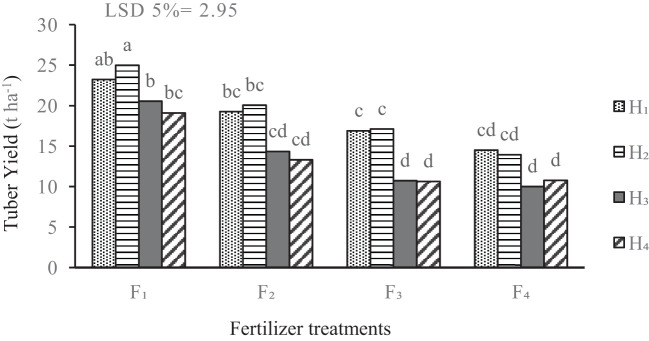
Mean comparison for the interaction of fertilizer treatments and weed management on potato yield. F_1_ complete chemical fertilizer (based on soil test results), F_2_ edible mushroom powder, F_3_ seaweed extract, F_4_ without fertilizer, H_1_ manual weeding, H_2_ metribuzin + paraquat herbicide, H_3_ biological herbicide, H_4_ without herbicide. Means followed by the same letter within a column are not significantly different according to the least significant difference (LSD) test.

Potato tuber yield was significantly influenced by the various fertilization and weed management strategies (**Table 2**). Combined treatment of complete chemical fertilizer and chemical herbicide application (F_1_H_2_) resulted in the highest yield, averaging 25 t.ha^-1^. Under the complete fertilizer treatment based on soil test (F_1_), the control group (no weed management) yielded notably higher than the other fertilization treatments. In the complete fertilization treatment, the yield from potatoes treated with biological herbicide (H_3_) did not significantly differ from that achieved with manual weeding (**Figure 3**).

The harvest index was also affected by fertilizer treatments; the complete fertilizer treatment yielded the highest index, averaging 80.96%, while the foliar seaweed extract treatment recorded the lowest at 77.88% (**Table 3**).

### Quality traits of potato tuber

**Figure 4:**
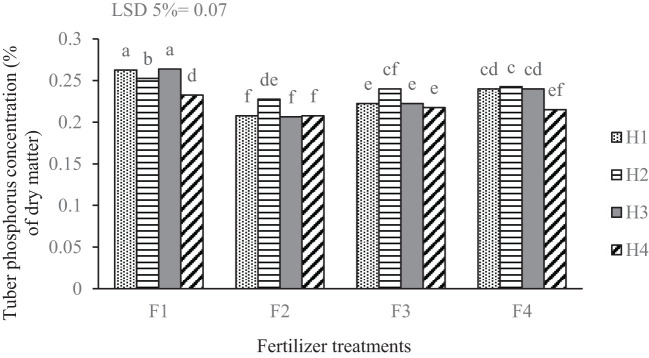
Mean comparison for interaction of fertilizer treatments and weed management on the phosphorus concentration of potato tuber. F_1_ complete chemical fertilizer (based on soil test results), F_2_ edible mushroom powder, F_3_ seaweed extract, F_4_ without fertilizer, H_1_ manual weeding, H_2_ metribuzin + paraquat herbicide, H_3_ biological herbicide, H_4_ without herbicide. Means followed by the same letter within a column are not significantly different according to the least significant difference (LSD) test.

The findings revealed that tuber phosphorus concentration was notably influenced by the interaction between fertilizer and weed management treatments (**Figure 4**). The combined treatments of F_1_H_1_ and F_1_H_3_ (both involving complete fertilizer treatment) led to the highest phosphorus concentrations in tubers. In contrast, the control treatment (without weed management) showed the lowest phosphorus concentration when fertilizer was applied based on soil tests. Furthermore, the foliar edible mushroom treatment consistently resulted in the lowest tuber phosphorus concentrations under various weed management methods, with the exception of manual weeding.

**Figure 5:**
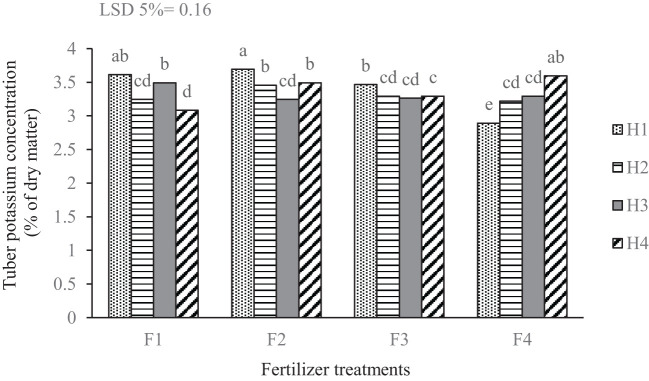
Mean comparison for the interaction of applied fertilizer treatments and weed management on the potassium concentration of potato tuber. F_1_ complete chemical fertilizer (based on soil test results), F_2_ edible mushroom powder, F_3_ seaweed extract, F_4_ without fertilizer, H_1_ manual weeding, H_2_ metribuzin + paraquat herbicide, H_3_ biological herbicide, H_4_ without herbicide. Means followed by the same letter within a column are not significantly different according to the least significant difference (LSD) test.

The results showed a significant variation in tuber potassium concentration based on the nutritional and weed management treatments (**Figure 5**). The highest potassium concentration was recorded in tubers treated with foliar application of edible mushrooms combined with mechanical weed management (hand weeding). However, this approach did not differ significantly in potassium levels from either the complete fertilizer treatment under mechanical weed management and the treatment without any fertilizer and weed management intervention. Conversely, the lowest potassium concentration occurred in tubers from the treatment lacking both fertilizer application and mechanical weed management.

**Figure 6:**
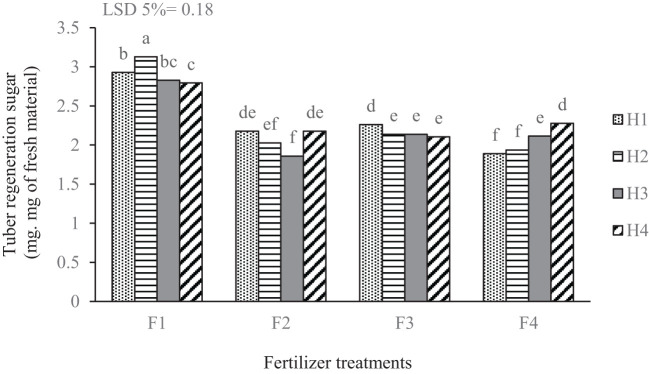
Mean comparison for the interaction of applied fertilizer treatments and weed management on the reducing sugars concentration of potato tuber. F_1_ complete chemical fertilizer (based on soil test results), F_2_ edible mushroom powder, F_3_ seaweed extract, F_4_ without fertilizer, H_1_ manual weeding, H_2_ metribuzin + paraquat herbicide, H_3_ biological herbicide, H_4_ without herbicide. Means followed by the same letter within a column are not significantly different according to the least significant difference (LSD) test.

The results indicated that reducing sugar concentrations in the tubers varied depending on the treatments applied. The highest concentration of reducing sugars was observed in the complete fertilizer treatment (based on soil analysis), significantly surpassing other nutritional treatments. Within the complete fertilization treatment (F_1_), chemical weed management yielded the highest reducing sugar concentration, while the lowest levels were found in both the control and biological herbicide treatments. The lowest reducing sugar concentrations occurred in the treatment without fertilizer, under both chemical and mechanical weed management. Furthermore, the foliar application of edible mushrooms combined with herbicide management produced a relatively low concentration of 1.85 mg g^-1^ of fresh weight, which did not differ significantly from the other low-concentration treatments. Foliar extracts of seaweed and edible mushrooms appear to moderate excessive increases in reducing sugars within the tubers (**Figure 6**).

**Figure 7:**
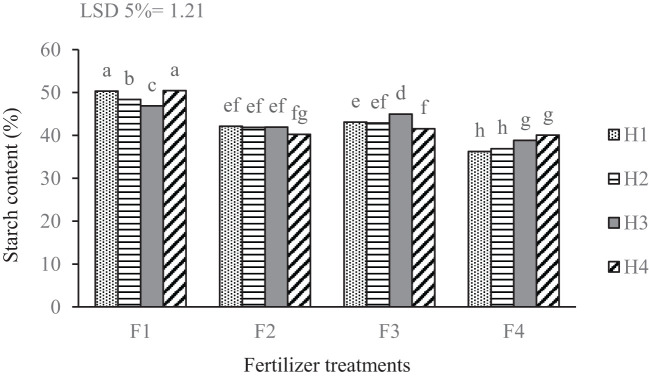
Mean comparison for the interaction of fertilizer treatments and weed management applied on the percentage of potato tuber starch content. F_1_ complete chemical fertilizer (based on soil test results), F_2_ edible mushroom powder, F_3_ seaweed extract, F_4_ without fertilizer, H_1_ manual weeding, H_2_ metribuzin + paraquat herbicide, H_3_ biological herbicide, H_4_ without herbicide. Means followed by the same letter within a column are not significantly different according to the least significant difference (LSD) test.

The complete fertilizer treatment, yielded the highest starch percentage among all fertilization levels, especially under manual weeding and in the absence of weed management (control). Conversely, the treatment incorporating a biological and chemical herbicide exhibited lower starch percentages. Foliar application of seaweed extract led to a significantly higher starch content than treatments with foliar edible mushroom extract and the control (no fertilizer). Notably, a marked increase in starch percentage was observed in the seaweed extract treatment when paired with biological herbicide management. The various treatments had significant effects on starch content, showing a substantial increase when herbicides were combined with biostimulants compared to the control (**Figure 7**).

**Figure 8:**
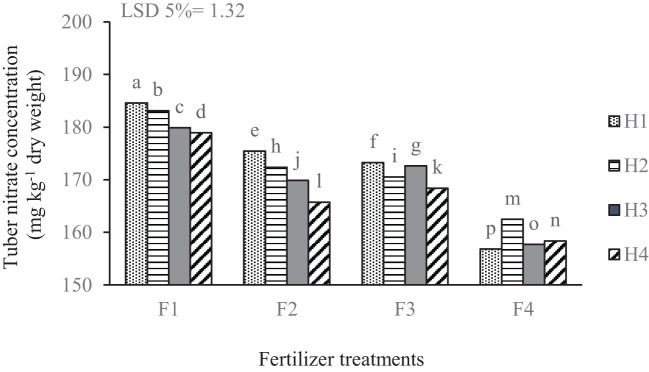
Mean comparison for the interaction of fertilizer treatments and weed management applied on the nitrate concentration of potato tuber. F_1_ complete chemical fertilizer (based on soil test results), F_2_ edible mushroom powder, F_3_ seaweed extract, F_4_ without fertilizer, H_1_ manual weeding, H_2_ metribuzin + paraquat herbicide, H_3_ biological herbicide, H_4_ without herbicide. Means followed by the same letter within a column are not significantly different according to the least significant difference (LSD) test.

The results revealed a significant variation in nitrate concentration in potato tubers across the different treatments (**Table 2**). Combined treatment of complete chemical fertilizer and manual weeding (F_1_H_1_) resulted in higher nitrate concentrations compared to other fertilization and weed management methods. In the no-fertilizer treatment, all weed management methods had lower tuber nitrate. As the herbicide treatments were shifted from manual weeding to various herbicides, a general trend of decreasing nitrate concentrations was observed within each fertilizer treatment group. The biological herbicide treatment consistently resulted in lower nitrate concentrations compared to the manual and chemical herbicide treatments across all fertilizer treatments (**Figure 8**).

## Discussion

### Yield components, yield and harvest index

The observed rise in stem number associated with chemical fertilizers and foliar amendents is primarily due to the nutrient availability that stimulates the growth of vegetative buds (Mystkowska et al., 2023). These findings are consistent with prior studies that demonstrate that nitrogen fertilizer application boosts the number of stems in potato plants (Oliveira, 2020).

Based on the results of the present study, the application of a biological herbicide before crop emergence (pre-emergence) has increased the number of main stems due to the absence of stress and negative effects on the sprouting of potato seed tubers, compared to other weed management treatments. Given that weeds often absorb nutrients more quickly and in greater quantities than crops (Kaur et al., 2018), it can be concluded that in the control treatment (without herbicide), one result of the competition between weeds and potatoes is a reduction in the number of main stems in the harvested produce. In fact, the lack of weed management in the control treatment has led to the depletion of nutritional resources, particularly nitrogen, and this has contributed to a decrease in the number of stems in the plant (Elrys et al., 2021).

In the present study, the number of tubers per unit area did not exhibit significant differences across all nutritional treatments (**Table 3**). However, the treatment without any fertilizer application yielded the lowest count. Clearly, the absence of various fertilizers fails to meet the crop’s nutritional requirements, which logically results in a decline in parameters such as the number of tubers per plant and per unit area.

The observation that all weed management treatments resulted in the highest tuber density supports the findings of Gugała et al. (2018), which indicated a direct correlation between effective weed management and improved tuber yields. Weeds can compete for space, reduce photosynthesis, and disrupt the balance between sources and sinks, potentially inhibiting tuber development by limiting the availability of photosynthetic materials (Chandel et al., 2022).

During this growth phase, the weight of the plant is affected by the concentrations of nutrients and the products of photosynthesis. The application of seaweed extract, which is rich in growth hormones, promotes improved nutrient absorption and distribution within the plant. Consequently, this can lead to a higher concentration of nutrients in the leaves, potentially resulting in an increased number of tubers per square meter (Mystkowska et al., 2023).

Despite the promising results regarding tuber count, tuber weight reflection was noteworthy. The complete fertilizer treatment optimally enhanced tuber weight, yielding an average of 82.7 g, with the foliar edible mushroom treatment producing significantly lighter tubers (**Table 3**). The presence of essential nutrients in the root zone (rhizosphere) is known to enhance plant metabolic processes, ultimately contributing to improved growth and yield (Bhaumik et al., 2024). However, an analysis of the average weight of tubers in the treatment lacking fertilizer application (control) reveals no statistically significant difference in average tuber weight when compared to the complete fertilizer treatment based on soil test results. Notably, the average tuber weight in the control group is even greater than that observed in two alternative nutritional treatments: foliar application of seaweed extract and foliar application of edible fungi. This apparent superiority in tuber weight can be attributed to the reduced number of tubers produced in the no-fertilizer treatment. Specifically, the lower tuber count in this control group results in a compensatory increase in individual tuber weight, although the total yield remains significantly lower compared to that of plants receiving the other nutritional treatments.

The results of this study highlight the combined application of complete chemical fertilizer and the use of a chemical herbicide (treatment F_1_H_2_) led to the highest yield. The superiority of the combined treatment can be attributed to the synergistic effects of nutrient availability and effective weed control. Chemical fertilizers, when properly applied based on soil tests, provide essential nutrients that promote optimal plant growth, while chemical herbicides effectively mitigate competition for resources from weeds (Singh et al., 2018). The enhanced yield in the F_1_H_2_ treatment underscores the importance of resource optimization in potato cultivation. Proper management of fertilizers ensures the balance of elements, which leads to increased yield and quality of agricultural products. Imbalanced application or overuse of chemical fertilizers can lead to nutrient deficiencies or toxicities, which may result in stunted growth, reduced yields, and inferior quality. Proper management practices can help prevent these problems while minimizing nutrient loss and pollution (McCauley et al., 2009). Moreover, the yield from potatoes treated with a biological herbicide (H_3_) under the complete fertilization treatment did not significantly differ from that achieved with manual weeding. This result suggests that biological herbicide strategies may be viable alternatives to traditional manual weeding, not only offering similar yields but also promising sustainability and reduced labor costs (Nath et al., 2024). Integrated weed management strategies that include both chemical inputs and the biological alternative can diversify control methodologies and reduce reliance on single-formula solutions (Müller-Schärer and Collins, 2020). Furthermore, high yields associated with comprehensive management strategies affirm the potential for improved efficiency in nutrient uptake and utilization (Nyiraneza and Snapp, 2007).

The complete fertilizer treatment indicated a robust harvest index of 80.96%, which aligns with previous studies that have highlighted the importance of balanced nutrient application in enhancing crop yield (Mystkowska et al., 2023). This suggests that the application of a complete fertilizer not only supports vegetative growth but also optimizes tuber development, which is critical for maximizing yield. On the other hand, the foliar edible mushroom powder treatment, which produced a nearly comparable harvest index of 80.08%, underscores that foliar spraying of organic fertilizers can serve as an effective complement to synthetic fertilizers. As noted by Das et al. (2024), the organic ammendents can improve nutrient availability, leading to enhanced crop yield and harvest index.

### Quality traits of tuber

The results of this study demonstrate the significant role of integrated management practices in influencing phosphorus concentration in tubers, with specific interactions between fertilizer types and weed management treatments proving particularly important. The observed effectiveness of the complete chemical fertilizer in conjunction with biological herbicides can be attributed to the improved soil nutrient availability and the mitigation of weed competition. Weeds are known to compete aggressively for nutrients, moisture, and light, which can lead to reduced nutrient uptake by the crop (Gallandt and Weiner, 2015). Additionally, the differential impact of complete chemical fertilizers and biological weed management indicates the importance of selecting appropriate agricultural practices tailored to specific crop needs and environmental conditions. Prior studies have documented that the method of nutrient application can significantly influence nutrient uptake, with a strong correlation between weed suppression and increased nutrient acquisition (Jain et al., 2022). Soliman et al. (2017) noted that potato plants treated with herbicides and hand hoeing exhibited greater phosphorus uptake in comparison to untreated plants, likely due to the reduction of competing weed species, allowing more phosphorus to be available for the treated plants. Zarzecka et al. (2010) demonstrated that phosphorus content and uptake are influenced by weed control methods, with manual weeding leading to greater phosphorus accumulation in potato tubers than chemical herbicide treatments. The application of foliar edible mushroom and seaweed extracts treatments resulted in consistently lower tuber phosphorus concentrations. This might suggest that while foliar applications can provide essential nutrients, they may not be sufficient to overcome the competitive disadvantage posed by weeds during critical growth stages.

The results of this study indicate that the highest potassium concentration in tubers was observed in plants subjected to a combination of foliar application of edible mushrooms and mechanical weed management through hand weeding. The use of organic extracts increases the efficiency of photosynthesis, improves the absorption of organic nutrients and water, and works to reduce transpiration. The mechanism of action of these extracts also affects the readiness of micro and macro elements, amino acids, vitamins and substances (Allen et al., 2001). Moreover, the manual removal of competition from weeds ensures that the plants have sufficient access to nutrients and water, potentially leading to enhanced tuber quality (Bručienė et al., 2022).

The highest reducing sugar concentrations were recorded under chemical weed management within the complete fertilization treatment (F_1_H_2_). This increase in sugar concentration resulting from chemical fertilizer application can be attributed to the fundamental roles of macro-nutrients (NPK) in the composition of photosynthetic pigment molecules and their influence on the assimilation rates of sugar precursors in tubers. These findings align with reports from Kolodziejczyk (2016) and Mijwel (2018). The relatively low concentration of reducing sugars observed with the foliar application of edible mushrooms and seaweed extract in all weed control systems. It seems that the application of foliar sprays using seaweed and edible mushroom extracts has prevented the excessive increase of reducing sugars in the tubers. It is likely that the compounds present in these extracts improve nutritional conditions and enzyme activity. In fact, as a result of spraying these compounds, the process of starch degradation into sugars has been delayed. The increase in soluble sugars in the tubers leads to an increased Maillard reaction during processing and reduces the quality of the produced products (Freitas et al., 2012).

The findings of this study highlight the significant effect of fertilization practices on tuber starch content, particularly when combined with different weed management strategies. The results indicate that plots treated with complete fertilizers yielded the highest starch percentages, particularly under conditions of manual weeding and control (no weed management). This suggests that optimal nutrient supply can enhance the growth conditions of the crops and improve starch synthesis, aligning with previous research that has established the positive correlation between nutrient availability and carbohydrate accumulation in plants (Zhang et al., 2021). In contrast, the treatment incorporating both biological and chemical herbicides resulted in lower starch percentages. Herbicides, while necessary for controlling weed populations, can have unintended effects on crop health if not properly balanced with fertilization strategies (El-Hadary and Chung, 2013). The observed increase in starch content from the seaweed extract treatment, compared to both the foliar edible mushroom extract and the control, emphasizes the unique benefits that seaweed may confer on plant health and productivity. Several mechanisms could explain the superior outcomes associated with seaweed extract. Seaweeds are known to contain a rich array of bioactive compounds, including phytohormones, amino acids, and micronutrients, which can enhance plant physiological processes (Ali et al., 2021b). The presence of auxins, cytokinins, and gibberellins in seaweed extracts has been linked to improved nutrient uptake and enhanced photosynthetic activity, both of which are critical for starch synthesis (Deolu-Ajayi et al., 2022). In contrast, while edible mushroom extract is also beneficial, it may lack the encompassing range of growth-promoting substances found in seaweed. Similarly, Zarzecka et al. (2021) have underscored the positive effects of biostimulants such as seaweeds on starch accumulation.

The treatment that involved complete chemical fertilizer paired with manual weeding (F_1_H_1_) produced significantly higher nitrate levels than all other combinations. The application of chemical inputs, particularly nitrogen fertilizers, appears to have resulted in elevated nitrate concentrations in treatments that utilized these fertilizers. This increase is likely a consequence of the rapid nutrient release, which exceeds the requirements and absorption capacity of the targeted crop (Liu et al., 2014). The observed trend of decreasing nitrate concentrations as weed management treatments transitioned from manual weeding to different chemical and biological herbicides suggests that mechanical disturbances, while temporarily effective in controlling weeds, may have led to a more significant release of nutrients from the soil, particularly nitrogen (Owen et al., 2006). Effective weed control treatments mitigate both below- and above-ground weed competition, which can hinder potato plant growth. By reducing this competition, potato plants can better leverage their photosynthetic capacity, ultimately increasing the synthesis, translocation, and accumulation of metabolites in the tubers, thereby improving yield and quality traits (Sharshar et al., 2015).

## Conclusion

In conclusion, the results of this study unequivocally demonstrate the critical influence of integrated nutrient management and weed control strategies on potato yield and quality traits. The significant interactions between nutritional and weed management treatments reveal how these factors collectively impact the growth dynamics of potato plants. Notably, the combined approach of complete chemical fertilizer applications and effective weed control—particularly through manual weeding—yielded the highest results in terms of total yield and nutrient concentrations. This affirms the necessity of a synergistic strategy for optimizing both crop performance and quality. The findings indicate that alternative treatments, such as foliar applications of seaweed and edible mushroom extracts, can serve as complementary tools in sustainable potato production systems. These treatments not only enhanced specific yield components but also moderated the adverse effects associated with traditional chemical inputs, such as excessive accumulation of reducing sugars and nitrate concentrations. Moreover, the positive outcomes associated with biological herbicides demonstrate their potential to provide a comparable yield to mechanical weeding while promoting environmentally sustainable practices.

## Data Availability

The raw data supporting the conclusions of this article will be made available by the authors, without undue reservation.
